# Right Ventricular Function Quantification in Takotsubo Cardiomyopathy Using Two-Dimensional Strain Echocardiography

**DOI:** 10.1371/journal.pone.0103717

**Published:** 2014-08-04

**Authors:** Felix Heggemann, Karsten Hamm, Joachim Brade, Florian Streitner, Christina Doesch, Theano Papavassiliu, Martin Borggrefe, Dariusch Haghi

**Affiliations:** 1 First Medical Department, University Medical Center Mannheim, University of Heidelberg, Mannheim, Germany; 2 Department of Cardiology, Center of Cardiovascular Medicine, Bad Neustadt, Germany; 3 Department of Biometrics and Statistics, University Medical Center Mannheim, University of Heidelberg, Mannheim, Germany; Stellenbosch University, South Africa

## Abstract

**Aims:**

This study sought to characterize global and regional right ventricular (RV) myocardial function in patients with Takotsubo cardiomyopathy (TC) using 2D strain imaging.

**Methods:**

We compared various parameters of RV and left ventricular (LV) systolic function between 2 groups of consecutive patients with TC at initial presentation and upon follow-up. Group 1 had RV involvement and group 2 did not have RV involvement.

**Results:**

At initial presentation, RV peak systolic longitudinal strain (RVPSS) and RV fractional area change (RVFAC) were significantly lower in group 1 (−13.2±8.6% vs. −21.8±5.4%, p = 0.001; 30.7±9.3% vs. 43.5±6.3%, p = 0.001) and improved significantly upon follow-up. Tricuspid annular plane systolic excursion (TAPSE) did not differ significantly at initial presentation between both groups (14.8±4.1 mm vs. 17.9±3.5 mm, p = 0.050). Differences in regional systolic RV strain were only observed in the mid and apical segments. LV ejection fraction (LVEF) and LV global strain were significantly lower in group 1 (36±8% vs. 46±10%, p = 0.006 and −5.5±4.8% vs. −10.2±6.2%, p = 0.040) at initial presentation. None of the parameters were significantly different between the 2 groups upon follow-up. A RVPSS cut-off value of >−19.1% had a sensitivity of 85% and a specificity of 71% to discriminate between the 2 groups.

**Conclusion:**

In TC, RVFAC, RVPSS, LVEF and LV global strain differed significantly between patients with and without RV dysfunction, whereas TAPSE did not. 2 D strain imaging was feasible for the assessment of RV dysfunction in TC and could discriminate between patients with and without RV involvement in a clinically meaningful way.

## Introduction

In 1991 Dote et al. [1] described a novel syndrome named Takotsubo Cardiomyopathy (TC) consisting of transient left ventricular apical dysfunction (“apical ballooning”), minimal elevation of myocardial enzymes in the absence of coronary artery disease and electrocardiographic ST-T segment changes [2].

In a previous work we characterized global and regional LV function in patients with TC using 2D strain echocardiography [2]. RV involvement is another known feature of TC, occurring roughly in one-quarter of patients [3]–[7]. Right ventricular involvement in patients with TC is correlated with more severe complications and longer hospitalisation and is an important prognostic factor of the clinical course [6]. Therefore, it is desirable to accurately assess RV function in this disease entity.

Estimation of RV function is challenging due to the complex RV geometry [8], [9]. Usage of TAPSE (tricuspid annular systolic excursion) for the diagnosis of RV dysfunction is limited, because hyperkinetic basal segments may compensate for hypokinetic midventricular- and apical parts. RVFAC (right ventricular fractional area change) measures RV function in a single plane and therefore may not be a reliable measure of global RV function. 2D strain is a novel method that allows for a segment-based measurement of myocardial deformation and may therefore have the potential to quantify RV dysfunction more precisely than conventional parameters of RV function.

To the best of our knowledge, detailed echocardiographic quantification of RV function in TC using 2D strain echocardiography has not been performed so far. Therefore, in this study we sought to analyse RV function by using this novel technique in addition to conventional parameters of RV function.

## Methods

### Study population

Twenty-eight patients with the clinical diagnosis of TC undergoing clinically indicated echocardiography were recruited for this study. Diagnosis of TC was based on the following criteria: (i) acute onset of apical and/or mid-ventricular wall-motion abnormalities of the left ventricle not confined to the vascular territory of a single major coronary artery, (ii) absence of severe coronary obstruction (>70%) or angiographic evidence of plaque rupture, (iii) new ECG abnormalities (either ST-segment elevation and/or T-wave inversion) or elevated cardiac troponin in the absence of phaechromocytoma and myocarditis. Depending on the additional presence of visually observed RV wall-motion abnormalities, patients were divided into two groups: fourteen patients with RV involvement (group 1) and fourteen without RV involvement (group 2).

### Echocardiography

Echocardiography of both groups was performed within 48 h of diagnosis and upon follow-up, using a Vivid 7 machine (GE Ultrasound, Horten, Norway) with a 2,5 MHz phased-array transducer. Standard echocardiographic images were obtained at end-expiratory apnoea. 2D strain analysis was performed offline using custom 2D strain imaging software (EchoPac, GE Ultrasound). The endocardial borders were traced at the end-systolic frame of the 2D images. The interactive software then automatically tracked myocardial motion and divided each image into six segments. For each of the six segments numerical and graphical displays of deformation parameters were generated, reflecting the average value for tracking all acoustic markers in each segment [10]. Longitudinal RV peak systolic strain (RVPSS) was acquired in the myocardium of the RV free wall from the apical four-chamber view that was modified, if necessary, to include the RV free wall. LV longitudinal peak systolic strain was acquired from apical two-, three- and four-chamber views. RVPSS was calculated as the average RV peak systolic longitudinal strain values of the basal, mid and apical RV free wall. RV fractional area change (RVFAC) and tricuspid annular plane systolic excursion (TAPSE) were assessed in the four-chamber view. LV ejection fraction (LVEF) was determined using Simpson's method. LV global longitudinal strain (LVGLS) was assessed from 3 apical views.

### Statistical analysis

Continuous variables are expressed as mean ± standard deviation. Continuous variables were compared using the two-sided Mann-Whitney U-test. A p-value <0.05 was considered statistically significant. Receiver operating characteristic (ROC) curves and Kendall-tau-b correlation coefficients were computed to assess the ability of RVPSS, RVFAC, TAPSE and LVGLS to predict RV involvement. Inter- and intraobserver correlation coefficients for strain measurements in our laboratory were 0.82 and 0.98, respectively [9].

## Results

The clinical and echocardiographic features of patients at initial presentation are shown in table 1. All patients were in sinus rhythm. Patients in both groups either had classical TC with apical involvement or mid-ventricular variant of TC, where apical wall-motion abnormalities are absent. None of the patients had the reverse variant of TC where LV dysfunction is confined to the base of the heart.

**Table 1:** Baseline clinical and echocardiographic characteristics for patients with TC with biventricular involvement (group 1) and only left ventricular involvement (group 2).

Parameters	group 1	group 2	p-value
Age (years)	68±8	70±12	0.265
Female, n (%)	12 (86)	14 (100)	0.541
Hypertension, n (%)	6 (43)	10 (71)	0.21
Diabetes, n (%)	2 (14)	4 (29)	0.541
Current smoker, n (%)	4 (29)	3 (21)	0.769
Hypercholesterolemia, n (%)	3 (30)	1 (8)	0.376
Pleural effusion (%)	5 (36)	0 (0)	0.003
Presenting symptom, n (%)			
*Angina*	6 (43)	11 (49)	0.114
*Dyspnoea*	6 (43)	2 (14)	0.210
*Other*	2 (14)	1 (8)	0.769
ECG upon presentation			
*ST elevation*	4 (29)	4 (29)	1.000
*T wave inversion*	12 (86)	11 (79)	0.769
Type TC, n (%)			
*Classical*	11 (79)	9 (64)	0.541
*Variant*	3 (21)	5 (36)	0.541
Peak troponin I (µg/l) [normal range: 0–0.4]	2.4±1.8	2.4±2.2	0.402
Triggering factor, n (%)			
*Physical stress*	7 (50)	2 (14)	0.114
*Emotional stress*	7 (50)	5 (36)	0.541
Strain obtainable in % of segments of the RV free wall (acute/follow-up)	81/76	83/71	

TC: Takotsubo cardiomyopathy, ECG: electrocardiogram, RV: right ventricular

**Figure 1:**
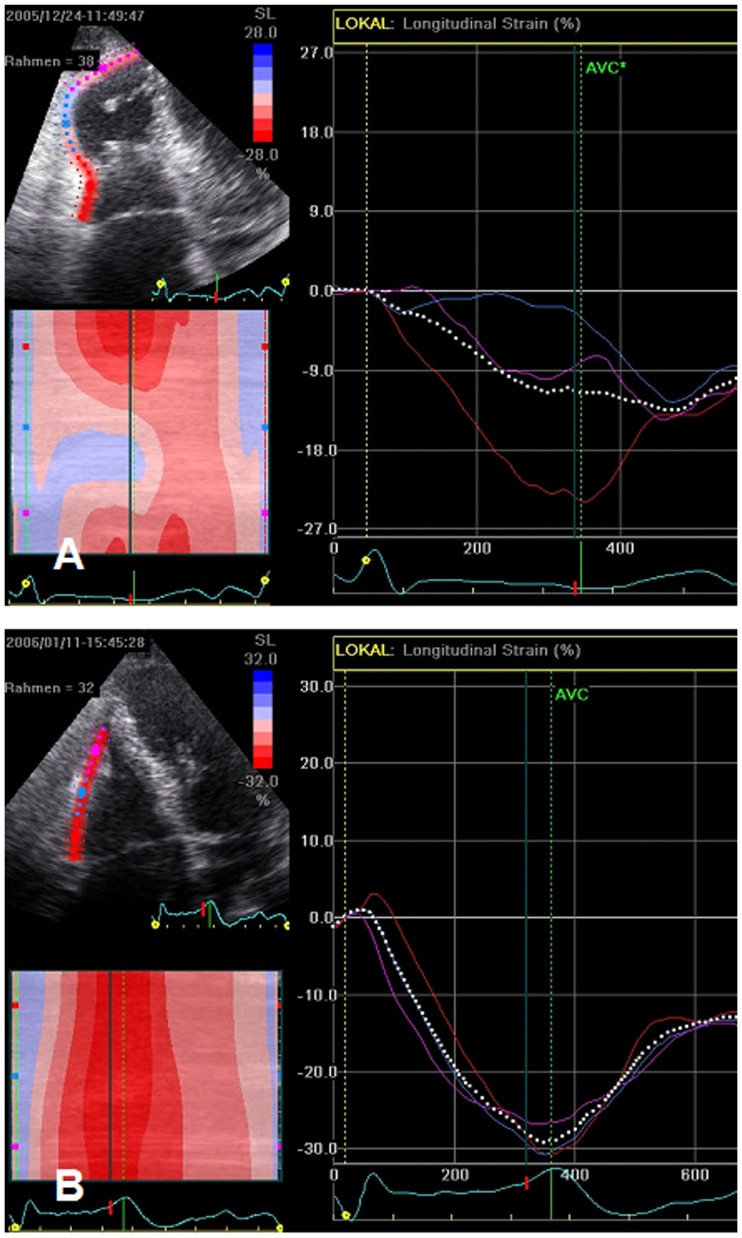
Right ventricular (RV) free wall systolic strain curves in a patient with RV involvement at baseline (A) and upon follow-up (B).

Follow-up examinations were performed in group 1 after 40±45 days and in group 2 after 68±170 days. Longitudinal peak systolic strain could be obtained in group 1 in 34 of 42 segments (81%) at baseline and in 32 of 42 segments (76%) upon follow-up (group 2, 35 of 42 segments (83%) at baseline and 30 of 42 segments (71%) upon follow-up). Figure 1 shows an example of RV free wall systolic strain in a patient with RV involvement at baseline (A) and upon follow-up (B). Table 2 shows regional RV strain values of both groups at baseline and upon follow-up. Regional longitudinal RV strain was significantly lower in apical and mid-ventricular segments at baseline in group 1 compared to group 2, and improved significantly in group 1 upon follow-up. RV strain was not significantly impaired in the basal segments at baseline.

**Table 2:** Right ventricular longitudinal strain (%) by segment in patients with Takotsubo cardiomyopathy with biventricular involvement (group 1) and only left ventricular involvement (group 2).

	group 1			group 2			
Segment	baseline	follow-up	p-value	baseline	follow-up	p-value	p-value**
apical free wall	−11.1±6.8	−22.1±8.5	**0.003**	−22.5±5.9	−26.1±8.3	0.244	**<0.001**
mid free wall	−13.5±11.4	−24.9±7.9	**0.020**	−25.3±6.5	−27.7±7.9	0.211	**0.005**
basal free wall	−16.6±14.1	−24.6±8.7	0.149	−25.5±6.9	−29.9±4.5	0.236	0.139

** group 1 compared to group 2 at baseline.

RVPSS and RVFAC were significantly lower in group 1 compared to group 2 at baseline, and improved significantly in group 1 but not in group 2 upon follow-up (table 3). TAPSE improved significantly in group 1 upon follow-up but was not significantly different at baseline compared to group 2. Upon follow-up, no significant differences could be found between both groups with regard to all 3 parameters. Differences in RVPSS were predominantly based on lower strain values in apical and mid-ventricular segments.

**Table 3:** Right and left ventricular echocardiographic parameters.

Parameters	group 1	group 2	p-value
TAPSE baseline (mm)	14.8±4.1*	17.9±3.5***	0.050
TAPSE follow -up (mm)	20.2±5.7**	20.3±4.0****	p = 0.072 ** vs. *: p = 0.010 **** vs. ***: p = 0.128
RVFAC baseline (%)	30.7±9.3*	43.5±6.3***	p = 0.001
RVFAC follow-up (%)	44.7±11.2**	39.6±7.3****	p = 0.169 ** vs. *: p = 0.006 **** vs. ***: p = 0.880
RVPSS baseline (%)	−13.2±8.6*	−21.8±5.4***	p = 0.001
RVPSS follow-up (%)	−24.1±6.6**	−26.0±7.2****	p = 0.378 ** vs. *: p = 0.002 **** vs. ****: p = 0.252
LVGLS baseline (%)	−5.5±4.8*	−10.2±6.2***	p = 0.040
LVGLS follow-up (%)	−14.1±2.8**	−18.1±2.6****	p = 0.378 ** vs. *: p <0.001 **** vs. ***: p = 0.001
Biplane ejection fraction (%) baseline	36±8 *	46±10 ***	p = 0.006
Biplane ejection fraction (%) follow-up	55±9 **	57±6 ****	p = 0.375 ** vs. *: p<0.001 **** vs.***: p = 0.007

TAPSE: tricuspid annular plane systolic excursion, RVFAC: right ventricular fractional area change, RVPSS: right ventricular peak systolic strain, LVGLS: left ventricular global strain.

*-**** indicate the statistically compared subgroups for each parameter.

**Figure 2:**
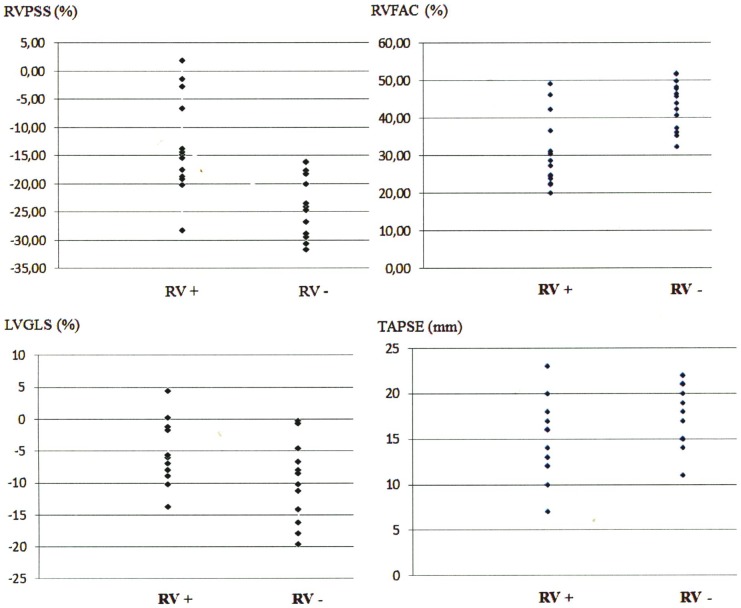
Distribution of RVPSS, TAPSE, RVFAC and LVGLS among patients with (RV+) and without (RV-) right ventricular involvement displayed by dotplots. RVPSS: right ventricular peak systolic strain, TAPSE: tricuspid annular plane systolic excursion, RVFAC: right ventricular fractional area change, LVGLS: left ventricular global strain.

**Figure 3:**
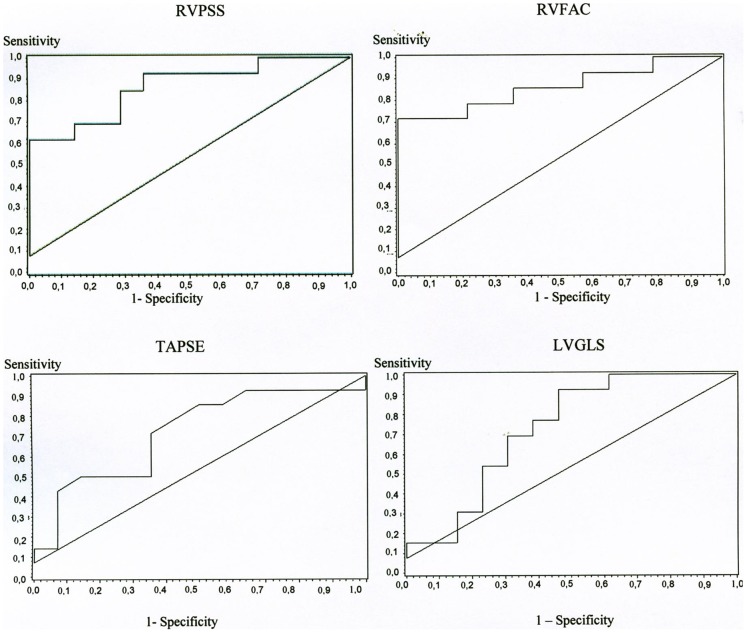
Correlation of RVPSS, RVFAC, TAPSE and LVGLS with occurrence of RV dysfunction displayed by receiver operating characteristic (ROC) curves. RVPSS: right ventricular peak systolic strain, TAPSE: tricuspid annular plane systolic excursion, RVFAC: right ventricular fractional area change, LVGLS: left ventricular global strain.

The dot plots in figure 2 display the distribution of RVPSS, TAPSE, RVFAC and LVGLS among the 2 groups. All 4 parameters were significantly correlated with RV involvement (table 4). ROC curves of these parameters are shown in figure 3. RVPSS had the best discriminatory value for differentiating between the 2 groups. RVPSS>−19.1% was 85% sensitive and 71% specific for predicting RV involvement. Pleural effusion (PE) occurred exclusively in group 1 (n = 5). Within group 1, patients with PE had significantly lower RVPSS compared to patients without PE (−4.65±6.3 vs.−18.46±4.5, p = 0.003). A RVPSS value >−14.8% indicated the occurrence of PE with a sensitivity of 87% and a specificity of 80%. At baseline, LVEF and LVGLS were significantly lower in group 1 patients compared to group 2 patients (table 3). Both parameters improved significantly upon follow-up in both groups. No significant differences could be found between both groups upon follow-up.

**Table 4:** Correlation of RVPSS, TAPSE, RVFAC and LVGLS with occurrence of RV- involvement in TC using Kendall-Tau-b correlation coefficient.

	Kendall-Tau-b correlation coefficient	p-value
RVPSS	0.490	0.004
RVFAC	−0.499	0.003
TAPSE	−0.379	0.025
LVGLS	0.329	0.048

RV: right ventricular, TC: Takotsubo cardiomyopathy, RVPSS: right ventricular peak systolic strain, RVFAC: right ventricular fractional area change, TAPSE: tricuspid annular plane systolic excursion, LVGLS: left ventricular global strain.

## Discussion

Assessment of RV function is one of the most challenging tasks in modern echocardiography. Subjective analysis is probably the most commonly used method for assessment of RV function. RV speckle tracking echocardiography (STE) is a novel method that allows quantitative assessment of RV function. Utility of RV STE both as a diagnostic and prognostic tool has been shown in various disease entities, including pulmonary hypertension (PH) [11], congenital heart disease [12], [13], heart failure [14] and cardiomyopathies [15].

RV involvement is a well-known feature of TC. Initial experience was confined to single cases [4], [5]. In 2006 Elesber et al. [6] reported on the Mayo Clinic experience in 25 consecutive patients. In their retrospective echocardiographic study, RV dysfunction was assessed visually and was found to be present in 8 of 25 patients. The authors found that RV dysfunction in TC indicated longer hospitalisation course, increased risk of hemodynamic instability, more severe congestive heart failure, and in some cases the requirement for intra-aortic balloon pump. They also found that the pattern of RV regional wall motion abnormalities was similar to the pattern of LV wall motion abnormalities.

Using cardiovascular magnetic resonance imaging Haghi et al. [16] were able to confirm most of these findings. RV involvement was present in roughly one-quarter of 34 patients and was associated with more depressed LV function. RV dysfunction was also indicative of extensive or bilateral pleural effusion.

The current study examined the utility of STE in the assessment of regional and global LV and RV function in TC. The importance of measuring RV longitudinal function is underlined by the fact that longitudinal shortening accounts for the majority of RV contraction in normal subjects and patients with PH [17]. We were able to measure longitudinal strain in 78% of all segments. According to our results impaired global RV function in TC seems to be predominantly caused by mid-ventricular and apical RV free wall strain reduction. This is consistent with previous studies using visual estimation of regional wall motion during echocardiography [6] and cardiovascular magnetic resonance imaging [16] in TC.

Previous studies in patients with heart failure [18], chronic PH [19], arrhythmogenic right ventricular disease [20] and in patients referred for cardiac surgery [21] have found that strain imaging is more precise in the quantification of RV function than the global parameters RVFAC and TAPSE. The complex shape of the right ventricle and the monoplane measurement are one reason why RVFAC has to be used with caution. Although RVFAC values were significantly different between patients with and without RV involvement in our study, the degree of overlap between both groups was high and no clinically useful cut-off value to separate between both groups could be established. More importantly, TAPSE which is believed to be a useful and simple tool for the assessment of RV function could not differentiate between patients with and without RV involvement. One reason for this observation could be that hypercontractility of basal segments, which is a well-known phenomenon during the acute phase of TC, could compensate for hypocontractility of the mid and apical segments, therefore, leading to similar values for TAPSE in both groups.

We found that RVPSS >−19.1% indicates RV involvement in patients with TC with a sensitivity of 85% and specificity of 71%. Previous studies have shown that RVPSS is superior to conventional parameters of RV function in various disease entities. For example, Teske et al. [22] found that a cut-off value of −18.1% in any RV segment could differentiate between patients with and without arrhythmogenic RV cardiomyopathy with a sensitivity and specificity of 91%. Ternacle et al. [21] observed that a cut-off value of −21% was an independent prognostic marker of mortality in patients referred for cardiac surgery. Guendouz et al. [18] found that in chronic heart failure patients with RVPSS >−21% were at greatest risk for worse outcome. Right heart failure is known to increase the risk of PE [24], [25]. In our assessment, PE was found only in the group of patients with RV involvement. A RVPSS of >−14.8% was a good indicator for the occurrence of PE (sensitivity 87%, specificity 80%). Although RVPSS is one of the most reliable parameters of RV function quantification, one limitation of this parameter is the fact that only the RV free wall, which is known to only contribute 80% of the RV stroke volume [18], is analysed.

All patients with RV involvement also had LV involvement. Cases of isolated RV TC have been published (25–27] but were not observed in our series. Patients of the present study had classical (apical) or variant (mid-ventricular involvement) forms of TC. RV-involvement occurred in both forms of TC.

It has been described that patients with RV involvement have higher degrees of LVEF reduction and longer hospitalization compared to patients without RV involvement [6], [7]. In our study RV involvement was associated with significantly lower LVEF and global LV strain which supports the hypothesis that these patients could have a more severe initial insult.

The pathophysiological mechanism underlying TC has not been clearly established. The biventricular involvement confirms the notion that TC is not a consequence of coronary artery occlusion [10], [16]. Although entirely speculative, differences in the distribution and/or density of RV adrenoceptors could be a potential explanation for RV involvement in TC.

### Limitations

There are limitations to this study that must be acknowledged. Because of the small number of patients the results should be regarded with caution. Some of the myocardial segments were uninterpretable due to signal noise. This was not always caused by poor acoustic windows. Tracking problems were particularly common in the LV and RV base.

## Conclusion

We conclude that 1) RV function quantification can be performed in patients with TC using 2D strain echocardiography. 2) Patients with TC who have RV involvement have significant impairment of several parameters of regional and global RV function. This is predominantly caused by dysfunction of RV mid and apical segments. 3) Patients with RV involvement have significantly lower LVEF and lower LV global strain at baseline which might be caused by a more severe initial insult. 4) A RVPSS cut off value of >−19.1% can differentiate between patients with and without RV dysfunction in a clinically meaningful way. 5) Pleural effusion was only found in patients with RV involvement and a RVPSS value >−14.8% indicated the occurrence of PE with good sensitivity and specificity.
